# Supplementary material for: Discontinuation rates and predictors of Medical Cannabis cessation for chronic musculoskeletal pain
Source: PLoS One. 2025 Aug 7;20(8):e0329897. doi: 10.1371/journal.pone.0329897 (PMC12331077; doi:10.1371/journal.pone.0329897)
Supplement: Attachment — Submitted filename: Discontinuation of medical cannabis.pdf [file pone.0329897.s001.pdf]

PLOS ONE

Discontinuation Rates and Predictors of Medical Cannabis Cessation for Chronic Musculoskeletal Pain

--Manuscript Draft--

Manuscript Number:	PONE-D-25-19383
Article Type:	Research Article
Full Title:	Discontinuation Rates and Predictors of Medical Cannabis Cessation for Chronic Musculoskeletal Pain
Short Title:	Medical Cannabis Discontinuation in Musculoskeletal Pain
Corresponding Author:	Sina Ramtin<br>The University of Texas at Austin Dell Medical School<br>Austin, Texas UNITED STATES OF AMERICA
Keywords:	cannabis; Musculoskeletal Pain; Discontinuation Rate; Pain Management
Abstract:	ABSTRACT<br>Background: Medical cannabis (MC) is increasingly used as a treatment for chronic musculoskeletal pain, but there is limited data on factors influencing its discontinuation. This study aims to investigate the rates of MC discontinuation and explore factors influencing it in patients with chronic musculoskeletal pain.<br>Methods: A retrospective analysis was conducted on 78 patients certified for medical cannabis over a 2 year period. Patient demographics, pain origin, and Global Physical Health (GPH) and Global Mental Health (GMH) scores were collected at baseline. Discontinuation rates were measured at three-month and one-year follow-up. Statistical analyses, including Fisher's Exact Test and two-sample t-tests, were performed to assess associations between variables and discontinuation.<br>Results: The overall discontinuation rate of MC use was 57.9% at one year, with 44.7% of patients discontinuing within the first three months. Older age was significantly associated with higher discontinuation rates. Pain origin categories did not significantly differ between those who discontinued and continued MC use, although a higher proportion of patients in the discontinued group reported low back pain. No significant differences were observed in baseline GPH and GMH scores between the two groups.<br>Conclusions: This study demonstrates a high discontinuation rate of MC use in patients for chronic musculoskeletal pain. The absence of significant differences in pain origin or baseline health scores suggests that factors beyond pain location or general health may contribute to the decision to discontinue MC. Further research is needed to explore the long-term effects of MC on pain management and patient outcomes.
Order of Authors:	Mohammad Khak
	Sina Ramtin
	JULIET CHUNG
	ASIF M. ILYAS
	ARI M. GREIS
Additional Information:	
Question	Response
**Financial Disclosure**<br><br>Enter a financial disclosure statement that describes the sources of funding for the work included in this submission. Review the submission guidelines for detailed requirements. View published research articles from *PLOS ONE* for specific examples.	The author(s) received no specific funding for this work.

Powered by Editorial Manager® and ProduXion Manager® from Aries Systems Corporation

This statement is required for submission and **will appear in the published article** if the submission is accepted. Please make sure it is accurate.<br><br>**Funded studies**<br>Enter a statement with the following details:<br>• Initials of the authors who received each award<br>• Grant numbers awarded to each author<br>• The full name of each funder<br>• URL of each funder website<br>• Did the sponsors or funders play any role in the study design, data collection and analysis, decision to publish, or preparation of the manuscript?<br><br>Did you receive funding for this work?	
**Competing Interests**<br><br>Use the instructions below to enter a competing interest statement for this submission. On behalf of all authors, disclose any competing interests that could be perceived to bias this work—acknowledging all financial support and any other relevant financial or non-financial competing interests.<br><br>This statement is required for submission and **will appear in the published article** if the submission is accepted. Please make sure it is accurate and that any funding sources listed in your Funding Information later in the submission form are also declared in your Financial Disclosure statement.<br><br>View published research articles from *PLOS ONE* for specific examples.	The authors have declared that no competing interests exist.

Powered by Editorial Manager® and ProduXion Manager® from Aries Systems Corporation

**NO authors have competing interests**<br><br>Enter: *The authors have declared that no competing interests exist.*<br><br>**Authors with competing interests**<br><br>Enter competing interest details beginning with this statement:<br><br>*I have read the journal's policy and the authors of this manuscript have the following competing interests: [insert competing interests here]*<br><br>* typeset	
**Ethics Statement**<br><br>Enter an ethics statement for this submission. This statement is required if the study involved:<br><br>• Human participants<br>• Human specimens or tissue<br>• Vertebrate animals or cephalopods<br>• Vertebrate embryos or tissues<br>• Field research<br><br>Write "N/A" if the submission does not require an ethics statement.<br><br>General guidance is provided below. Consult the submission guidelines for detailed instructions. **Make sure that all information entered here is included in the Methods section of the manuscript.**	Consent was obtained or waived by all participants in this study. Thomas Jefferson University Office of Human Research issued approval 19D.159. This study was approved by the IRB of Thomas Jefferson University, Philadelphia, PA.

Powered by Editorial Manager® and ProduXion Manager® from Aries Systems Corporation

**Format for specific study types**<br><br>**Human Subject Research (involving human participants and/or tissue)**<br>• Give the name of the institutional review board or ethics committee that approved the study<br>• Include the approval number and/or a statement indicating approval of this research<br>• Indicate the form of consent obtained (written/oral) or the reason that consent was not obtained (e.g. the data were analyzed anonymously)<br><br>**Animal Research (involving vertebrate animals, embryos or tissues)**<br>• Provide the name of the Institutional Animal Care and Use Committee (IACUC) or other relevant ethics board that reviewed the study protocol, and indicate whether they approved this research or granted a formal waiver of ethical approval<br>• Include an approval number if one was obtained<br>• If the study involved *non-human primates*, add *additional details* about animal welfare and steps taken to ameliorate suffering<br>• If anesthesia, euthanasia, or any kind of animal sacrifice is part of the study, include briefly which substances and/or methods were applied<br><br>**Field Research**<br><br>Include the following details if this study involves the collection of plant, animal, or other materials from a natural setting:<br>• Field permit number<br>• Name of the institution or relevant body that granted permission	
**Data Availability**<br><br>Authors are required to make all data underlying the findings described fully available, without restriction, and from the time of publication. PLOS allows rare exceptions to address legal and ethical concerns. See the PLOS Data Policy and FAQ for detailed information.	Yes - all data are fully available without restriction

A Data Availability Statement describing where the data can be found is required at submission. Your answers to this question constitute the Data Availability Statement and **will be published in the article**, if accepted.<br><br>**Important:** Stating 'data available on request from the author' is not sufficient. If your data are only available upon request, select 'No' for the first question and explain your exceptional situation in the text box.<br><br>Do the authors confirm that all data underlying the findings described in their manuscript are fully available without restriction?	
**Describe where the data may be found in full sentences. If you are copying our sample text, replace any instances of XXX with the appropriate details.**<br><br>• If the data are **held or will be held in a public repository**, include URLs, accession numbers or DOIs. If this information will only be available after acceptance, indicate this by ticking the box below. For example: *All XXX files are available from the XXX database (accession number(s) XXX, XXX.)*.<br>• If the data are all contained **within the manuscript and/or Supporting Information files**, enter the following: *All relevant data are within the manuscript and its Supporting Information files.*<br>• If neither of these applies but you are able to provide **details of access elsewhere**, with or without limitations, please do so. For example:<br><br>*Data cannot be shared publicly because of [XXX]. Data are available from the XXX Institutional Data Access / Ethics Committee (contact via XXX) for researchers who meet the criteria for access to confidential data.*<br><br>*The data underlying the results presented in the study are available from (include the name of the third party*	Data cannot be shared publicly because of patient confidentiality and institutional restrictions. Data are available from the Thomas Jefferson University Institutional Review Board through the corresponding author for researchers who meet the criteria for access to confidential data.

and contact information or URL).<br>• This text is appropriate if the data are owned by a third party and authors do not have permission to share the data.<br><br>* typeset	
Additional data availability information:	

Powered by Editorial Manager® and ProduXion Manager® from Aries Systems Corporation

Manuscript

Click here to access/download;Manuscript;MC Compliance draft 74 Final draft.docx

TITLE:

Discontinuation Rates and Predictors of Medical Cannabis Cessation for Chronic Musculoskeletal Pain

AUTHORS:

(1) MOHAMMAD KHAK, MD, MPH [1]

(2) SINA RAMTIN, MD [1]

(3) JULIET CHUNG, MD [1]

(4) ASIF M. ILYAS, MD, MBA [1,2,3]

(5) ARI GREIS, DO [1,2,3]

STUDY LOCATION:

Department of Medical Cannabis, Rothman Orthopaedic Institute, Philadelphia, PA, USA

AFFILIATIONS:

1 – Rothman Institute Foundation for Opioid Research & Education, Philadelphia, PA, USA

2 – Rothman Orthopaedic Institute at Thomas Jefferson University, Philadelphia, PA, USA

3 – Drexel University College of Medicine, Philadelphia, PA, USA

ACKNOWLEDGEMENTS:

This study was supported through funding from the Rothman Institute Foundation for Opioid Research & Education in Philadelphia, PA, USA.

ABSTRACT

Background: Medical cannabis (MC) is increasingly used as a treatment for chronic musculoskeletal pain, but there is limited data on factors influencing its discontinuation. This study aims to investigate the rates of MC discontinuation and explore factors influencing it in patients with chronic musculoskeletal pain.

Methods: A retrospective analysis was conducted on 78 patients certified for medical cannabis over a 2 year period. Patient demographics, pain origin, and Global Physical Health (GPH) and Global Mental Health (GMH) scores were collected at baseline. Discontinuation rates were measured at three-month and one-year follow-up. Statistical analyses, including Fisher's Exact Test and two-sample t-tests, were performed to assess associations between variables and discontinuation.

Results: The overall discontinuation rate of MC use was 57.9% at one year, with 44.7% of patients discontinuing within the first three months. Older age was significantly associated with higher discontinuation rates. Pain origin categories did not significantly differ between those who discontinued and continued MC use, although a higher proportion of patients in the discontinued group reported low back pain. No significant differences were observed in baseline GPH and GMH scores between the two groups.

Conclusions: This study demonstrates a high discontinuation rate of MC use in patients for chronic musculoskeletal pain. The absence of significant differences in pain origin or baseline health scores suggests that factors beyond pain location or general health may contribute to the decision to discontinue MC. Further research is needed to explore the long-term effects of MC on pain management and patient outcomes.

Keywords: Cannabis; Musculoskeletal Pain; Discontinuation Rate; Pain Management

INTRODUCTION

Chronic musculoskeletal pain is a prevalent condition that affects the quality of life of millions of individuals worldwide. Despite advancements in pharmacologic and non-pharmacologic therapies, many patients continue to experience persistent pain and disability. As a result, there has been growing interest in alternative treatments, including medical cannabis (MC), for managing chronic pain. The therapeutic application of MC has gained global attention due to its potential to alleviate pain. Cannabis and its derivatives are believed to act on CB1 receptors in the brain, modulating pain signals, and CB2 receptors in the dorsal root ganglion, which influence pain integration in nerve pathways (1). While MC has shown promise in providing significant relief from chronic pain, often surpassing 50% improvement compared to a placebo (2), its effects on cognition, particularly executive function, memory, and attention, remain a concern. Short-term memory impairment is commonly associated with MC use, and chronic use can exacerbate cognitive deficits, leading to slower processing and reduced attention. Furthermore, long-term and early-age use of MC has been linked to neurocognitive deficits, with neuroimaging studies showing reduced hippocampal volume and density (3).

Despite the growing acceptance of MC as a therapeutic option for chronic musculoskeletal pain, significant gaps remain in understanding its long-term efficacy. While some patients report significant pain relief, others experience dissatisfaction, intolerance, or prefer more definitive treatments, such as surgery or joint injections (4, 5). These factors contribute to the complexity of integrating MC into routine clinical practice. Furthermore, limited research exists on the reasons for MC discontinuation, which is crucial for optimizing its clinical use.

This study aims to address these gaps by investigating the discontinuation of medical cannabis in patients with chronic musculoskeletal pain. We focus on factors such as age, pain origin, and baseline health status. Understanding the predictors of discontinuation will provide valuable insights into the factors that influence patient decisions regarding MC use, helping guide clinical recommendations and treatment strategies.

METHODS

Institutional review board approval was obtained before the initiation of data collection. Physicians participating in MC certification completed a mandatory four-hour continuing medical education course and sought accreditation from the Pennsylvania Department of Health. The patient certification process involved verifying the presence of one of the 23 state-approved medical conditions, confirming Pennsylvania residency, and reviewing the patient's mental health and pain management history. Eligible individuals underwent a chart review to identify any significant history of substance abuse or severe mental health disorders, with those patients being excluded from the certification process. Additionally, a review of controlled substance use history was conducted using the Pennsylvania Prescription Drug Monitoring Program. If a patient was currently prescribed opioids, their prescribing physician was consulted to discuss the integration of MC into the treatment plan. During the certification visit, patients were provided comprehensive information on the chemical composition of MC, available delivery methods, optimal dosing guidelines, and recommended formulations by the certifying physicians. Upon certification, patients were issued a medical cannabis identification card by the Pennsylvania Department of Health, enabling them to purchase cannabis at authorized dispensaries within the state.

The medical records of 78 patients diagnosed with chronic musculoskeletal noncancer pain who were certified for MC at the Medical Cannabis Department of the Rothman Orthopaedic Institute between October 2022 and December 2024 were analyzed. These patients, previously described in a prior publication on short-term outcomes (6), were reviewed for a minimum follow-up period of one year to assess MC discontinuation rates. Following their initial MC certification, patients were scheduled to attend follow-up visits at three months and subsequently on an annual basis. To further evaluate the continuity of MC use among these patients, we accessed the Pennsylvania Medical Marijuana Program's patient registry. This registry allows practitioners to verify patients' certification statuses and identify whether they have obtained recertifications through other healthcare providers. By cross-referencing our patient cohort with this database, we determined the recertification status of each individual, providing insights into their ongoing engagement with MC treatment across different medical practices.

Patient demographics were obtained through electronic medical records for all patients. At the initial visit, we collected data on pain in specific regions, including low back, neck, shoulder, and foot and ankle pain, as well as the Patient-Reported Outcomes Measurement

Information System (PROMIS) global health scale. Due to the variability in the site of musculoskeletal pain, we utilized global pain outcome measures rather than site-specific disability measures. The PROMIS global health scale provided two key scores: Global Physical Health (GPH) and Global Mental Health (GMH) quality of life (QoL) T-scores. Statistical analysis for GPH and GMH was conducted using the Shapiro-Wilk test to assess normality. The relationship between explanatory variables and GPH and GMH was tested using T-tests, as the outcome variables were normally distributed. A p-value of <0.05 was considered statistically significant in the analysis.

RESULTS

The Follow-Up Outcomes

The 3-month and 1-year follow-up data was available for the 76 of 78 patients. By the first 3-month period, 44.7% (34 patients) had discontinued MC. Reasons for discontinuation included dissatisfaction with MC, joint or epidural injections, and surgical intervention. After the 3-month follow-up, an additional 13.2% (10 patients) ceased MC use, resulting in a total discontinuation rate of 57.9% (44 patients) at 1 year. The 1-year study cohort of MC tolerance thus consisted of 32 patients among which 11 patients (14.5%) continued treatment with other physicians outside of our practice (Figure 1).

Figure 1. Patient Retention and Cannabis Use Over Time

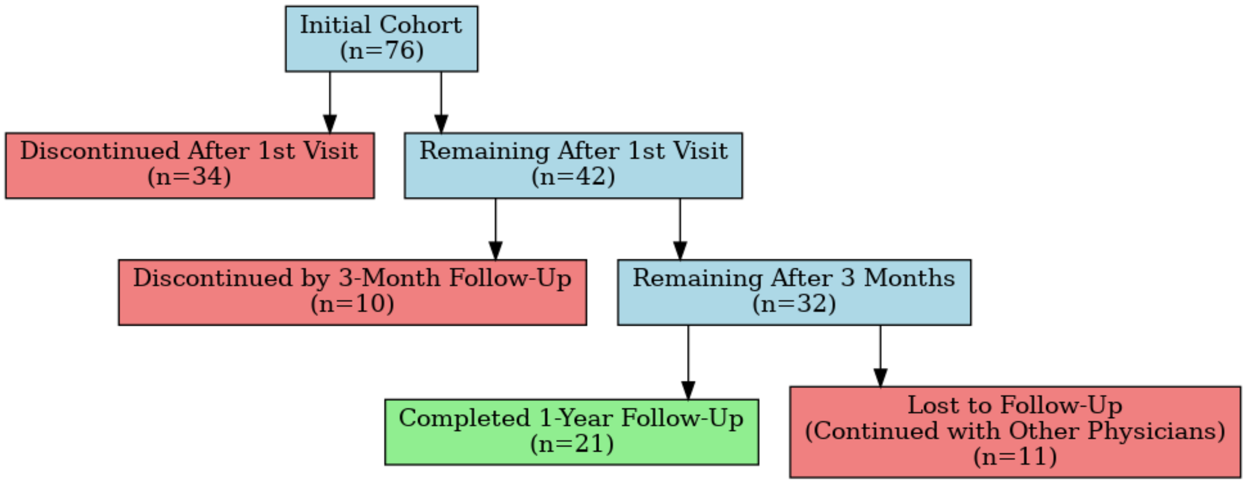

We compared the characteristics of patients who continued MC use after one year with those who discontinued its use. There was a significant difference in age between the two groups (p = 0.04) (Figure 1). Patients who discontinued MC were older, with a mean age of 71.5 ± 9.8 years compared to those who continued MC, who had a mean age of 64.5 years ± 10.2. There was no significant difference in insurance type between the two groups. Of the 44 patients who discontinued MC, 47.7% were on Medicare and 52.3% were on HMO/PPO insurance, while 37.5% of those who continued MC were on Medicare and 62.5% were on HMO/PPO. Race distribution did not differ significantly between the groups with 97.7% of patients who discontinued MC being White compared to 90.6% of those who continued MC. Of the patients who discontinued MC, 61.4% were female and 38.6% were male, compared to 65.6% female and 34.4% male in the group that continued MC with no significant difference (Tabe 1).

Regarding health outcomes, there were no significant differences in GPH or GMH scores between the two groups. The mean GPH score for patients who discontinued MC was 43.6 ± 9.8, while those who continued MC had a mean GPH score of 43.9 ± 10.2. Similarly, the mean GMH score for patients who discontinued MC was 46.7 ± 7.5, compared to 47.2 ± 9.3 for those who continued MC.

Table 1. Bivariate Analysis of Discontinued vs. Continued Medical Cannabis Use

**Variable**	**Continued MC (n = 32)**	**Discontinued MC (n = 44)**	**P-value**
**Age (Median)**	64.5 years	71.5 years	0.04
**Insurance Type**			0.3
- Medicare	37.5%	47.7%	
- HMO/PPO/O	62.5%	52.3%	
**Race**			0.1
- White	90.6%	97.7%	
- Other	9.4%	2.3%	
**Gender**			0.7
- Female	65.6%	61.4%	

- Male	34.4%	38.6%	
**GPH Score**	43.9 ± 10.2	43.6 ± 9.8	0.8
**GMH Score**	47.2 ± 9.3	46.7 ± 7.5	0.7

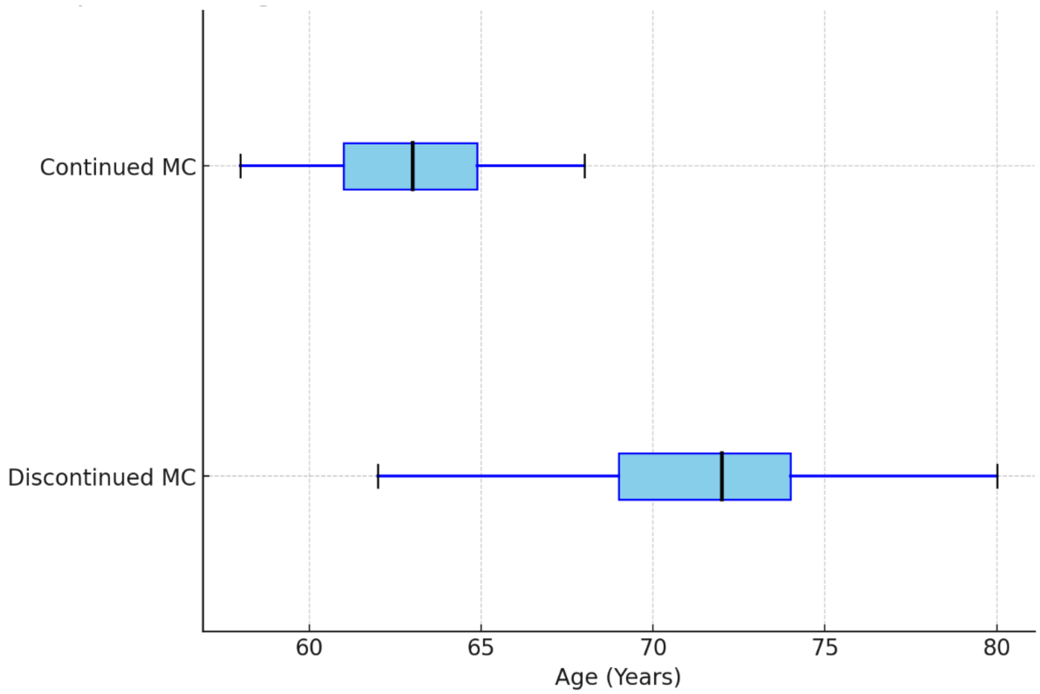

Figure 1. Comparison Of Age Between Patients Who Discontinued vs. Continued Medical Cannabis (MC) Use after 1 Year.

The distribution of pain origin was analyzed between patients who discontinued MC and those who continued its use. The following categories were reported: low back pain, neck pain, Foot/Ankle pain, shoulder pain, and knee pain. We combined all categories associated with low back pain (low back pain only, low back and neck pain, low back and Foot/Ankle pain, and low back and shoulder pain) and compared the occurrence of low back pain between the two groups. There was no significant difference in the occurrence of low back pain between the 30 patients (68.2%) who discontinued MC and 18 patients (56.3%) who continued MC after 1 year ($p = 0.3$). For all other pain origin categories, Fisher's Exact Test was applied, and none of the results were

statistically significant (all p-values > 0.05) suggesting that the distribution of pain origins between the two groups remained relatively similar across all categories (Figure 2).

Figure 2. Pain Origin Distribution Between Discontinued and Continued Medical Cannabis Users

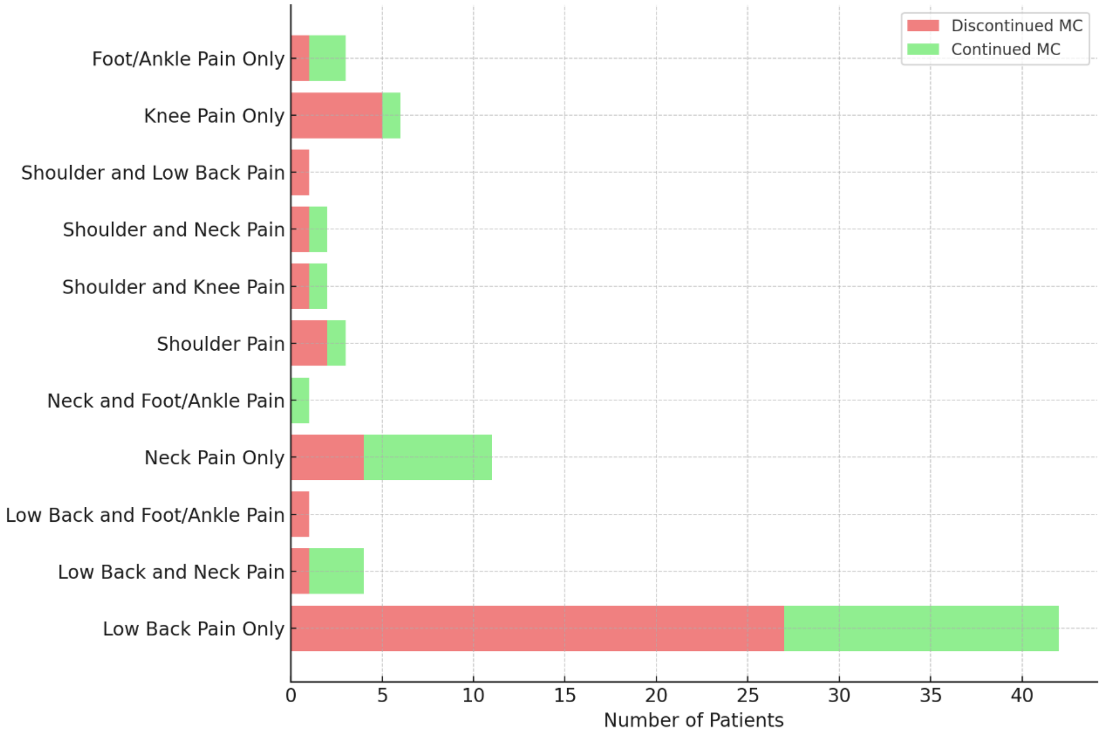

DISCUSSION

This study aimed to investigate the rate of cessation and factors influencing the discontinuation of MC use in patients with chronic musculoskeletal pain. A key finding in this study was the high discontinuation rate, with 57.9% of patients ceasing MC use within one year. This suggests that a large proportion of patients may experience dissatisfaction, intolerance, or prefer alternative treatments such as surgery or joint injections for managing chronic musculoskeletal pain. However, it is important to note that 42.1% of patients either continued MC use or sought recertification, indicating that a subset of patients perceive sustained benefits from MC. These results are consistent with previous studies, which have shown mixed responses to MC treatment in chronic pain patients. While some patients report significant relief, others may not find sufficient therapeutic benefit, leading to early discontinuation (7, 8).

By the three-month follow-up, 44.7% had already discontinued MC, reflecting a considerable early drop-off. This early discontinuation could point to initial expectations not being met, potential side effects, or insufficient symptom relief, which are common reasons for discontinuation in medical treatments. The relatively high early discontinuation rate indicates that MC may not provide immediate or sustained relief for all patients and highlights the need for better patient selection and management strategies in the early stages of treatment. To address the high discontinuation rates observed in medical cannabis treatment for chronic pain, recent studies have utilized machine learning models to predict therapy dropout and identify influential factors. Visibelli et al (9) employed a random forest classifier to predict dropout in a cohort of 542 patients undergoing cannabis-based treatment for chronic pain. The study found that high final VAS scores and elevated THC dosages were the most significant predictors of discontinuation, while factors like baseline therapeutic benefits and CBD dosages were associated with improved adherence.

The age of patients was found to be significantly associated with the likelihood of discontinuing MC. Older patients were more likely to cease MC use, which aligns with findings from other studies suggesting that older adults may be more cautious in using alternative therapies like MC due to concerns about long-term effects or a preference for more conventional treatments (10). One possible explanation for the higher discontinuation rates observed in the elderly population is the presence of more advanced musculoskeletal conditions, which may not respond well to medical cannabis. As patients age, chronic pain often becomes more complex and severe, leading them to seek more definitive treatments such as joint replacements or surgical

interventions. Furthermore, the progression of chronic diseases and the presence of comorbidities in older adults may also influence their decision to stop MC, as they may be more inclined to rely on treatments with well-established outcomes while younger individuals tend to continue exploring newer treatment options, including MC. Haug et al. (11) examined cannabis use patterns and motives among medical cannabis dispensary patients of different age groups. They suggest that age-related factors may influence medical cannabis use patterns and the likelihood of discontinuation, which is consistent with our observation that older patients were more likely to discontinue MC treatment.

The origin of pain was also assessed to determine if certain pain conditions influenced MC discontinuation rates over a one-year period. While no significant differences were observed between pain origin categories, we did combine all categories related to low back pain and found that a higher proportion of patients in the Discontinued MC group reported low back pain (68.18%) compared to the Continued MC group (56.25%). Although no statistical significance was reached, this difference suggests that low back pain, as a common and complex chronic condition, may contribute to patients' decisions to discontinue MC. The higher prevalence of low back pain in the Discontinued MC group may reflect the difficulties in managing pain of this nature with MC alone, as patients with chronic low back pain often seek more definitive treatments, such as joint injections or surgical interventions. However, the absence of significant findings points to the multifactorial nature of treatment adherence, where factors beyond pain location, such as psychological, social, and healthcare system-related variables, likely play a more substantial role in the decision to discontinue treatment. Previous studies have suggested that pain-related factors, including the chronicity and intensity of pain, are often associated with patients' decisions to either persist with or discontinue treatment (12-14).

The distribution of other pain origins, such as neck pain, shoulder pain, and knee pain, did not reveal significant differences between the two groups. These findings suggest that, overall, pain origin does not appear to be a determining factor in whether patients continue or discontinue MC use. This supports the hypothesis that MC may be used as an adjunctive treatment for multiple pain conditions, with its effectiveness varying based on individual patient responses rather than specific pain etiologies. However, this does not negate the importance of individualized treatment plans for patients with complex, multidimensional pain, where factors such as the patient's

psychological status, comorbidities, and social support systems are equally important considerations in determining treatment adherence.

Baseline GPH and GMH scores were measured but did not show significant differences between patients who discontinued and those who continued MC use. This suggests that at the time of MC certification, the two groups did not differ substantially in their overall physical or mental health status. However, it is important to recognize that these scores reflect only the baseline health status and do not capture the potential changes in physical and mental health outcomes over time as a result of MC use. Since MC is primarily used to manage chronic pain, it is likely that over time, changes in pain levels and functional status may influence GPH and GMH scores, potentially leading to improved or worsened outcomes that could affect a patient's decision to continue or discontinue treatment (15, 16). Future studies should examine how these scores evolve throughout the course of MC treatment to provide more comprehensive insights into the impact of MC on both physical and mental well-being. This study has several limitations. First, the retrospective nature of the analysis and the relatively small sample size may limit the generalizability of our findings. The sample was also limited to a specific cohort of patients who sought treatment at a single Medical Cannabis Department, which may not be representative of the broader population of individuals using MC for chronic pain management. Additionally, the study did not collect detailed data on the severity of pain, the level of functional impairment, or the quality of life of patients after one year, which may have provided additional insights into why some patients chose to discontinue MC. Future prospective studies with larger, more diverse populations and more comprehensive data collection on these variables would help to clarify the factors influencing MC use and discontinuation.

CONCLUSION

This study highlights the importance of discontinuation rate of MC use in patients with chronic musculoskeletal pain, with over half of patients discontinuing treatment within one year. Age was identified as a key factor influencing discontinuation, with older patients being more likely to stop using MC. While pain origin did not significantly affect discontinuation rates, a higher proportion of patients in the discontinued group reported low back pain, suggesting that the complexity of managing chronic pain conditions with MC alone may contribute to treatment cessation. Additionally, baseline Global Physical Health and Global Mental Health scores did not

show significant differences between the two groups, indicating that overall health status at the time of MC certification did not influence the decision to discontinue or continue treatment. These findings suggest that while MC may offer benefits for some patients, further research is needed to better understand the long-term effects of MC on pain management and patient satisfaction, as well as the factors influencing treatment adherence.

REFERENCES

1. Turgeman I, Bar-Sela G, editors. Cannabis use in palliative oncology: a review of the evidence for popular indications. Int Conf Med Cannabis; 2017.
2. Mücke M, Phillips T, Radbruch L, Petzke F, Häuser W. Cannabis-based medicines for chronic neuropathic pain in adults. Cochrane database of systematic reviews. 2018(3).
3. Urits I, Charipova K, Gress K, Li N, Berger AA, Cornett EM, et al. Adverse effects of recreational and medical cannabis. Psychopharmacology bulletin. 2021;51(1):94.
4. Furrer D, Kroger E, Marcotte M, Jauvin N, Belanger R, Ware M, et al. Cannabis against chronic musculoskeletal pain: a scoping review on users and their perceptions. J Cannabis Res. 2021;3(1):41.
5. Whiting PF, Wolff RF, Deshpande S, Di Nisio M, Duffy S, Hernandez AV, et al. Cannabinoids for Medical Use: A Systematic Review and Meta-analysis. JAMA. 2015;313(24):2456-73.
6. Chung J, Mahmoud Y, Ramtin S, Uhler G, Ilyas AM, Greis A. Understanding the Orthopedic Conditions for Which Patients Are Seeking Medical Cannabis Certification. Cureus. 2024;16(1).
7. Notcutt W, Price M, Miller R, Newport S, Phillips C, Simmons S, et al. Initial experiences with medicinal extracts of cannabis for chronic pain: results from 34 'N of 1' studies. Anaesthesia. 2004;59(5):440-52.
8. Harris D, Jones RT, Shank R, Nath R, Fernandez E, Goldstein K, et al. Self-reported marijuana effects and characteristics of 100 San Francisco medical marijuana club members. J Addict Dis. 2000;19(3):89-103.
9. Visibelli A, Finetti R, Roncaglia B, Poli P, Spiga O, Santucci A. Predicting therapy dropout in chronic pain management: a machine learning approach to cannabis treatment. Frontiers in Artificial Intelligence. 2025;8:1557894.
10. Silbaugh K. Medical cannabis and the age of majority. BUL Rev. 2021;101:1155.
11. Haug NA, Padula CB, Sottile JE, Vandrey R, Heinz AJ, Bonn-Miller MO. Cannabis use patterns and motives: A comparison of younger, middle-aged, and older medical cannabis dispensary patients. Addictive behaviors. 2017;72:14-20.
12. Lynch ME, Ware MA. Cannabinoids for the Treatment of Chronic Non-Cancer Pain: An Updated Systematic Review of Randomized Controlled Trials. J Neuroimmune Pharmacol. 2015;10(2):293-301.
13. Stockings E, Campbell G, Hall WD, Nielsen S, Zagic D, Rahman R, et al. Cannabis and cannabinoids for the treatment of people with chronic noncancer pain conditions: a systematic review and meta-analysis of controlled and observational studies. Pain. 2018;159(10):1932-54.
14. Nugent SM, Morasco BJ, O'Neil ME, Freeman M, Low A, Kondo K, et al. The Effects of Cannabis Among Adults With Chronic Pain and an Overview of General Harms: A Systematic Review. Ann Intern Med. 2017;167(5):319-31.
15. Chung J, Mahmoud Y, Ramtin S, Uhler G, Ilyas AM, Greis A, et al. Understanding the Orthopedic Conditions for Which Patients Are Seeking Medical Cannabis Certification. Cureus. 2024;16(1).

16. Greis A, Larsen E, Liu C, Renslo B, Radakrishnan A, Wilson-Poe AR. Perceived efficacy, reduced prescription drug use, and minimal side effects of cannabis in patients with chronic orthopedic pain. Cannabis and cannabinoid research. 2022;7(6):865-75.
