# Supplementary material for: Discontinuation rates and predictors of Medical Cannabis cessation for chronic musculoskeletal pain
Source: PLoS One. 2025 Aug 7;20(8):e0329897. doi: 10.1371/journal.pone.0329897 (PMC12331077; doi:10.1371/journal.pone.0329897)
Supplement: Attachment — Submitted filename: long-term, stable access to study data contact.docx [file pone.0329897.s003.docx]

Dear Editor-in-Chief,

Thank you for your follow-up regarding the data availability policy. We appreciate the importance of ensuring long-term, stable access to study data.

To align with PLOS ONE’s Data Policy, we confirm that requests for access to the minimal dataset used in this study can be directed to the following non-author institutional contact:

**Name:** Lilly Caso
**Title:** Research Administrative
**Institution:** Rothman Institute Foundation for Opioid Research & Education
**Email:** [Lilly.Caso@rothmanopioid.org](mailto:Lilly.Caso@rothmanopioid.org)
**Phone (if applicable):** [optional]
**Webpage :** **https://www.rothmanopioid.org/**

This contact will manage all external data access requests in compliance with our institutional policies. The dataset will be securely archived by the Rothman Institute Foundation to ensure long-term preservation and availability.

Please let us know if you require any additional documentation.

Sincerely,

Sina Ramtin
