# Supplementary material for: Discontinuation rates and predictors of Medical Cannabis cessation for chronic musculoskeletal pain
Source: PLoS One. 2025 Aug 7;20(8):e0329897. doi: 10.1371/journal.pone.0329897 (PMC12331077; doi:10.1371/journal.pone.0329897)
Supplement: Attachment — Submitted filename: point by point response 2nd.docx [file pone.0329897.s004.docx]

Dear Editor-in-Chief of PLOS ONE,

We sincerely thank you for the opportunity to revise and resubmit our manuscript titled:
**"Discontinuation Rates and Predictors of Medical Cannabis Cessation for Chronic Musculoskeletal Pain."**

We appreciate the thoughtful comments provided by the editorial team and peer reviewers. We have revised the manuscript accordingly and addressed each point as detailed below. All changes have been incorporated into the main text per PLOS ONE guidelines.

Please find our detailed responses below:

**Editorial concerns:**

1. “We note that there were multiple versions of [ Response to Reviewers ] in your submission's file inventory. Please double check your submission file inventory and let us know if any files appear to be outdated or absent.”

**Response:** The second file was from the previous revision, now removed.

2. “Thank you for including your ethics statement on the online submission form:
     "Consent was obtained or waived by all participants in this study. Thomas Jefferson University Office of Human Research issued approval 19D.159. This study was approved by the IRB of Thomas Jefferson University, Philadelphia, PA.". To help ensure that the wording of your manuscript is suitable for publication, would you please also add this statement at the beginning of the Methods section of your manuscript file. “

**Response**: appropriate wording was added to the beginning of the method section as: “Patient outcome measures in this study were derived from the Rothman Orthopaedic Cannabis Data Repository (ROCDR), which was established with approval from the Institutional Review Board at Thomas Jefferson University, Philadelphia, Pennsylvania (protocol number 19D.159). Consent was obtained or waived by all participants in this study.”

**Reviewer#2 Comments:**

1. “scores were collected at baseline”. Recommend substituting Baseline with before intervention.
   **Response:**
   Corrected. The term “baseline” has been replaced with “before intervention” throughout the manuscript.
2. “Executive function” includes Working Memory and selective attention
   **Response:**
   We have revised the manuscript to remove specific mentions of “working memory” and “selective attention” under “executive function” to improve clarity and accuracy.
3. “history of substance abuse or severe mental health disorders”. Substance abuse is a mental health disorder when it meets DSM-V criteria
   **Response:**
   Thank you for the clarification. The sentence has been updated to: “significant history of substance abuse or any other mental health disorders” to align with DSM-V definitions.
4. Include PROMIS 10 questionnaires using 5-point Likert scales. Rating is “limited to the past 7 days” before intervention.
   **Response:**
   We have added a reference to the questionnaire, which is available online at https://www.healthmeasures.net/.
5. “There was a significant difference in age between the two groups” – Statistically significant
   **Response:**
   We have updated the sentence to read: “There was a statistically significant difference in age between the two groups.”
6. Reference for “advanced musculoskeletal conditions which may not respond well to medical cannabis”
   **Response:**
   A supporting reference has been added to the manuscript to substantiate this statement.
7. “Advanced musculoskeletal conditions in the elderly” such as degenerative changes (radiculopathies, stenosis) or chronic wear and tear (OA, tendinopathies etc)
   **Response:**
   We have incorporated these specific examples—radiculopathies, stenosis, osteoarthritis (OA), and tendinopathies—into the manuscript to provide clarity.
8. “That study.” Please reference the study here
   **Response:**
   The study has been referenced as citation [15], and the sentence has been rephrased accordingly.
9. “due to its retrospective design”. Does 2-year follow-up after an intervention (MC) make it a prospective rather than a retrospective study?
   **Response:**
   To eliminate confusion, we have removed this sentence from the manuscript.
